# INSIGHTS FROM A HAPF ALUMNUS ON AGING POLICY ADVOCACY IN SOCIAL WORK

**DOI:** 10.1093/geroni/igad104.0026

**Published:** 2023-12-21

**Authors:** Nancy Kusmaul

**Affiliations:** University of Maryland Baltimore County, Baltimore, Maryland, United States

## Abstract

“Once a fellow, always a fellow” and “Networks get things done”. The Health and Aging Policy Fellows Program has brought together experts in aging for more than ten years for policy education and fellowships, assembling an alumni network of more than one hundred and fifty fellows. Fellow alumni are social work scholars, physicians, nurses, gerontologists, sociologists, and many more. Each of these alumni completed one or more placements in policy making in congress, federal agencies, and advocacy organizations. Some have remained in those spaces while others are academics, state and local officials, and corporate leaders. They continue to advocate for and shape policy in aging in those positions. Told from the perspective of a non-residential program alumnus who is a mid-career faculty member in social work, this presentation will discuss the lessons learned during the fellowship, how the fellowship shaped future policy work, and present insights on how social workers can engage in policy advocacy in aging through the fellowship.

